# Ascites in a Young Woman: A Rare Presentation of Eosinophilic Gastroenteritis

**DOI:** 10.1155/2018/1586915

**Published:** 2018-05-10

**Authors:** Carina Santos, Francisco Morgado, Celestina Blanco, João Parreira, João Costa, Lara Rodrigues, Luís Marfull, Patrícia Cardoso

**Affiliations:** Department of Internal Medicine, Radiology, University Hospital Center “Sousa Martins”, Guarda, Portugal

## Abstract

**Introduction:**

Eosinophilic gastroenteritis (EGE) is a rare idiopathic disease that can affect one or more organs of the digestive tract. It has an estimated incidence of 1–20 cases per 100,000 patients. Klein et al. classified EGE into 3 subtypes: predominant mucosal, muscular, or subserosal.

**Clinical Case:**

We report a case of a 32-year-old woman, who presented with diffuse abdominal pain, nausea, postprandial infarction, diarrhea, and moderate ascites of three-week evolution. The rest of physical examination did not show alterations. The past medical history was unremarkable. Laboratory test results revealed peripheral blood eosinophilia. Abdominal CT scan revealed diffuse and concentric parietal thickening of the distal 2/3 of esophagus, moderate volume ascites, and small bowel wall thickening and distension on the left quadrants. The paracentesis revealed 93.3% of eosinophils. The colon biopsies evidenced an increase in the number of eosinophils. Secondary causes of eosinophilia were excluded. The patient was treated with oral prednisolone 40 mg/day with immediate clinical and analytical improvement.

**Conclusion:**

Eosinophilic gastroenteritis is a rare condition with a nonspecific and highly variable clinical presentation, which requires a high level of clinical suspicion. It is a diagnosis of exclusion. Secondary causes of eosinophilia such as intestinal tuberculosis, parasitosis, and malignant neoplasms should be excluded.

## 1. Introduction

Eosinophilic gastroenteritis (EGE) is a rare idiopathic disease which can affect one or more organs of the digestive tract, including the stomach, small intestine, and less often the colon. Only 280 cases were described between 1937 and 2006 [1]. The estimated incidence of EGE remains relatively rare with 1–20 cases per 100,000 patients.

Although it may manifest from childhood to adulthood, the peak incidence occurs between the 3rd and 5th decades of life, with a slight predominance in males (3 : 2) [2]. The etiology and pathogenesis of EGE have not yet been established. The mechanisms responsible for GI tract infiltration by eosinophils remains controversial. However, several studies have shown that half of the patients with EGE presented a previous history of atopy. In addition, Immunoglobulin E (IgE) levels are elevated in some patients [3]. The diagnosis is suggested by clinical history and peripheral eosinophilia and confirmed by eosinophilic infiltration of the wall of the digestive tract [4], in the absence of known causes of eosinophilia [5].

In 1970, Klein et al. classified EGE into three types depending on the depth of eosinophilic infiltration: predominant mucosal, muscular, and subserosal.

The mucosal layer disease is the most frequent subtype and presents with nonspecific symptoms such as a general abdominal pain, nausea, diarrhea and vomiting which may mimic other gastrointestinal diseases; in the most severe form patients may present with blood loss in stools, iron deficiency anemia, or protein-losing enteropathy. The muscular subtype, the second most frequently reported, is characterized by infiltration of eosinophils predominantly in the muscle layer, causing bowel wall thickening and, consequently, symptoms of intestinal obstruction [2]. The subserosal subtype occurs in only 10% of the cases [5]; it presents typically with isolated abdominal ascites or ascites accompanied by symptoms of the mucosal or muscular forms; the hallmark of this subtype is marked eosinophilia in the ascitic fluid; frequently, this subtype has the best response to corticosteroids [2]; according to the guideline published in 2014 by the Japanese Ministry of Health, Labor and Welfare, as negative endoscopic mucosal biopsies do not definitively rule out muscular or subserosal EGE, laparoscopic full-thickness biopsy is necessary to establish the diagnosis [6].

Matsushita et al. studied a Japanese, a Japanese American, and a Caucasian group living in Hawaii, in what concerns the normal number of eosinophils in the gastrointestinal tract. Their results on the Japanese surgical biopsy samples showed a significant increase in the number of eosinophils from the esophagus to the right colon (mean ± SD/mm: 0.07 ± 0.43 for the esophagus, 12.18 ± 11.39 for the stomach, and 36.59 ± 15.50 for the right colon), compared with a decrease in the left colon (8.53 ± 7.83). Investigation using surgical samples showed that the distribution patterns in the gastrointestinal tract were very similar among the 3 ethnic groups, and there were no significant differences in the number of eosinophils among these groups, except in the esophageal epithelium. Their data suggest that a cutoff value for eosinophil counts, when rendering a diagnosis of eosinophilic gastrointestinal tract disease, should be individualized to the different biopsy sites. [7]. However, further studies needed to generalize these results among other geographic areas and ethnic groups.

Many authors agree that, in order to confirm the histological diagnosis, at least 15–20 eosinophils/high power microscopic fields are considered necessary [8].

Corticosteroids are effective in 80–100% of cases, although there are no randomized controlled trials for EGE treatment. Second-line therapies include mast cell stabilizers (sodium cromoglycate), antihistamines, leukotriene antagonists (montelukast), diet (elimination of milk, soy, wheat, eggs, peanuts, and seafood), immunotherapy (desensitization), immunomodulators (azathioprine and 6-mercaptopurine), mepolizumab, tumor necrosis factors inhibitors (e.g., infliximab), and IgE monoclonal antibody (omalizumab) [1].

The oral prednisolone dose most frequently administered is 40 mg/day for 7–14 days, followed by a progressive dose reduction. In some cases, maintenance treatment with low doses (5 to 10 mg a day) is necessary [9].

## 2. Clinical Case

A 32-year-old Caucasian woman, with no relevant medical history, was admitted to the Emergency Department due to generalized abdominal pain, nausea, postprandial infarction, diarrhea, and enlargement of abdominal perimeter, lasting 3 weeks. She reported an aggravation in the last 3 days. She denied other symptoms, including fever, night sweats, weight loss, arthralgia, or skin rash, as well as history of atopic diseases, food allergies, raw fishing eating, or recent medication intake.

**Figure 1:**
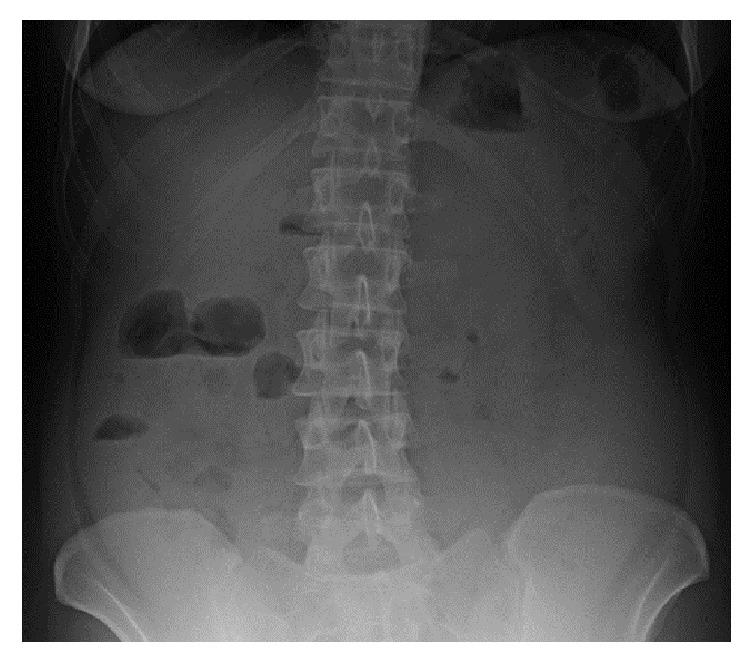
X-ray showing levels in the upper right quadrant of the abdomen.

On examination she had painful and distended abdomen. The cardiopulmonary examination was normal. Blood tests revealed leukocytosis [13360/microliter (*μ*l)] with 36.5% of eosinophils (4880/*μ*l). The abdomen X-ray showed levels in the upper right quadrant (Figure 1).

**Figure 2:**
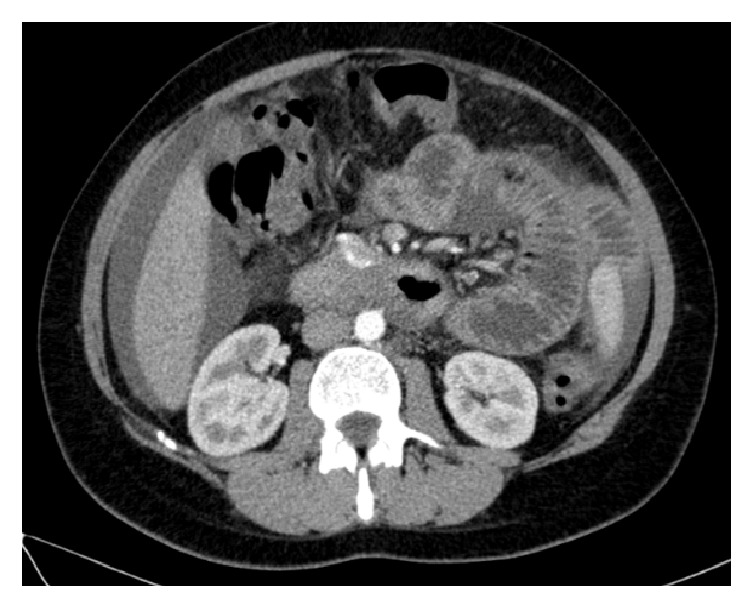
CT scan showing ascites and thickening and distension of small bowel loops.

Abdominal CT scan revealed diffuse and concentric parietal thickening of the distal two-thirds of esophagus, moderate volume ascites, and small bowel wall thickening with distension on the left quadrants (Figure 2)

**Figure 3:**
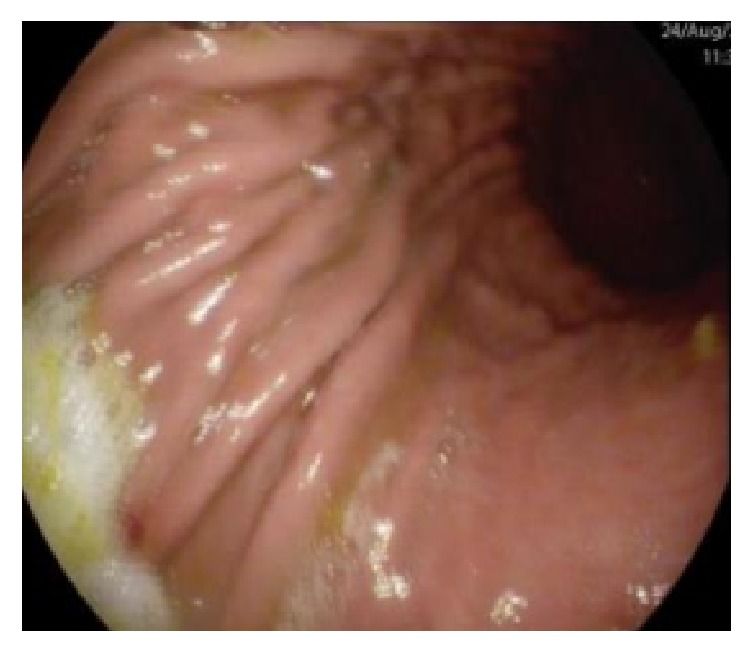
Greater curvature of the stomach.

**Figure 4:**
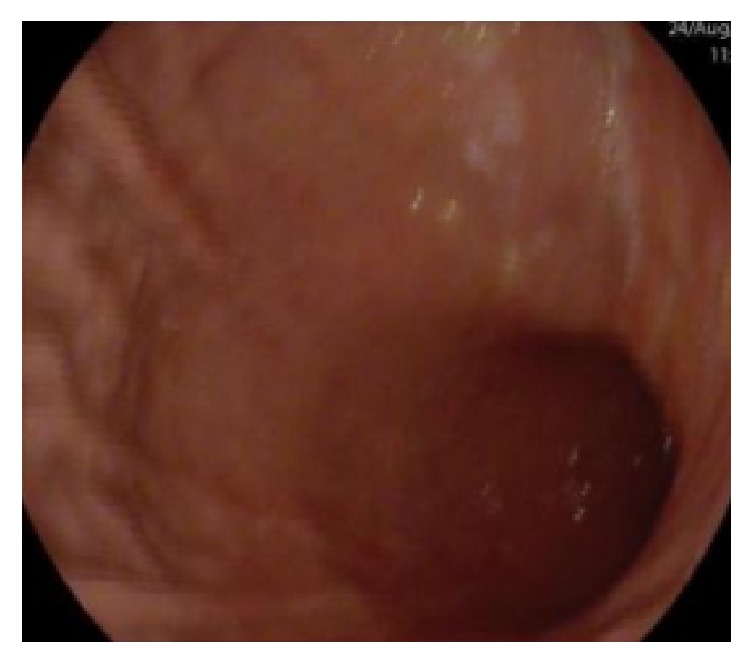
Gastric antrum and body.

The gastroscopy showed a “peptic ring at 40 cm and hyperemia of the body and antrum mucosa”; no biopsies were taken (Figures 3 and 4). She was admitted to the Internal Medicine Department for complementary study. The abdominal ultrasound showed several dilated loops in the small bowel, with significant reduction of their peristalsis, compatible with intestinal subocclusion. The parasitological stool exam was negative for eggs, cysts, and parasites. Serum IgE level was normal (83.8 KU/L).

The patient underwent an ultrasound-guided paracentesis. Results showed 6912 leukocytes/mm3, of which 93.3% were eosinophils (6450/*μ*l), without malignancy; laboratory testing of the ascitic fluid for bacterial culture and tuberculosis was negative.

**Figure 5:**
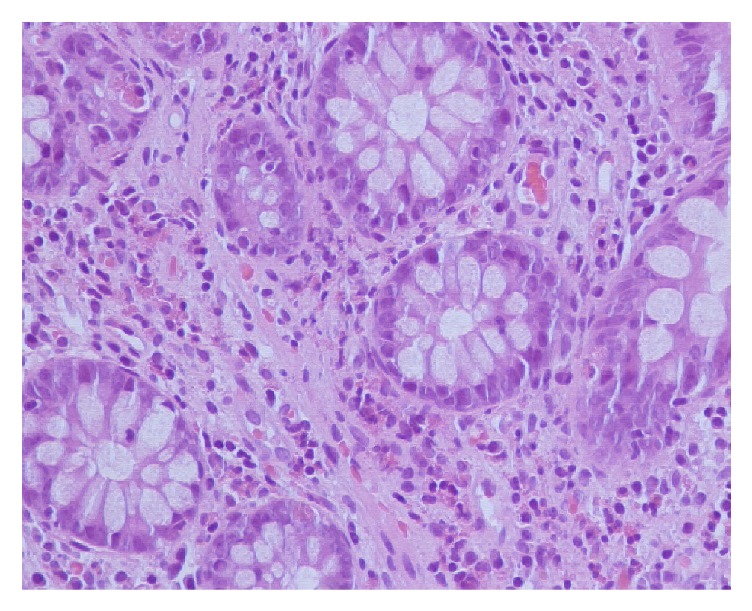
Colon biopsy with >20 eosinophils/field.

Colonoscopy showed a congestive and petechial ileocecal valve and hypotrophic mucosa with evidence of submucosal circulation, suspected of microscopic colitis. Colon biopsies showed mucosa with nodular lymphoid hyperplasia and increased number of eosinophils/high power microscopic fields in left colon (20–24/high power microscopic fields) (Figure 5).

The constellation of clinical, analytical, and histopathological results suggested the diagnosis of eosinophilic gastroenteritis.

The patient was started on 40 mg of oral prednisolone/day, with clinical and analytical rapid improvement (decrease of 5130 eosinophils/uL to 100 eosinophils/uL with 2 doses of prednisolone). To date, the patient stopped taking corticosteroids and remains asymptomatic.

## 3. Discussion

EGE is a rare medical condition that presents with nonspecific clinical symptoms. The diagnosis requires a high level of clinical suspicion. It is necessary to rule out other pathologies such as intestinal tuberculosis, parasitosis, and malignant neoplasms.

The clinical case presented refers to the rarest subtype of EGE which makes the diagnostic challenge even more difficult.

An early diagnosis and rapid introduction of corticosteroids may reduce the duration and severity of symptoms, thus reducing the morbidity of this disease. However, the clinician should keep in mind the possibility of a relapsing disease.

Given the limited number of EGE cases reported in the literature, further data are needed about the epidemiology, clinical and histological characteristics, and evolution of the disease.
